# Action video game training improves text reading accuracy, rate and comprehension in children with dyslexia: a randomized controlled trial

**DOI:** 10.1038/s41598-021-98146-x

**Published:** 2021-09-20

**Authors:** Jessica L. Peters, Sheila G. Crewther, Melanie J. Murphy, Edith L. Bavin

**Affiliations:** 1 Department of Psychology and Counselling, La Trobe University, Melbourne, VIC 3086 Australia; 2 Intergenerational Health, Murdoch Childrens Research Institute, Melbourne, Australia

**Keywords:** Attention, Dyslexia, Reading

## Abstract

Dynamic visual attention training using Action Video Games (AVGs) is a promising intervention for dyslexia. This study investigated the efficacy of 5 h (10 × 30 min) of AVG training in dyslexic children (aged 8–13) using ‘Fruit Ninja’, while exploring whether increasing attentional and eye movement demands enhanced AVG effectiveness. Regular (AVG-R; *n* = 22) and enhanced AVG training (AVG+; *n* = 23) were compared to a treatment-as-usual comparison group (*n* = 19) on reading, rapid naming, eye movements and visuo-temporal processing. Playing ‘Fruit Ninja’ for only 5 h significantly improved reading accuracy, rate, comprehension and rapid naming of both AVG groups, compared to the comparison group, though increasing attentional demands did not enhance AVG efficacy. Participants whose low contrast magnocellular-temporal processing improved most following training also showed significantly greater improvement in reading accuracy. The findings demonstrate a clear role for visual attention in reading and highlight the clinical applicability of AVGs as a fun, motivational and engaging intervention for dyslexia.

## Introduction

Dynamic visual attention training using Action Video Games (AVGs) produces significantly greater reading rate and fluency improvements in children with developmental dyslexia compared to non-AVG control interventions, with moderate-to-large effect sizes^1^. As AVGs do not involve any direct teaching of phonological, orthographic or reading skills, it is the attentional demands of playing AVGs that have been associated with these reading improvements^2^. AVGs have also been shown to benefit rapid automatic naming^3^, visual attention and phonological skills^2,4^. As such, AVG attention training may be more appealing for children with dyslexia and provide a more wide-spread benefit to reading subskills^5,6^ compared to current treatment options such as phonics training, which is efficacious in remediating single word identification skills i.e., irregular word accuracy and sight words^7^. However, further work is needed to determine which elements of dynamic visual attention contribute most to developing such skills, and to expand upon past findings by using different AVGs and training durations. These findings will help to inform planning for fast clinical and educational application.

### Attentional impairments in dyslexia

Reading is a dynamic process reliant on temporally and spatially accurate attention, with well-organized eye movements to shift attention. Those with dyslexia often demonstrate impairments in dynamic visual attention skills, including temporal processing^8^, distribution of attention^9^, ‘sluggish attentional shifting’^10^, and inefficient planning and coordination of rapid sequential eye movements during reading and non-reading tasks^11–13^. Such dynamic attention is predominantly driven by the faster visual magnocellular pathway that is responsive to high temporal and low spatial frequencies, and frequently found to be impaired in dyslexia^14,15^.

### The neuroscience of AVGs

Bavelier and Green^16^ suggest in a recent review that AVGs comprise two key drivers of learning and neural plasticity; (1) high demands on attentional control and (2) reward-driven motivated behaviour^16^. By definition, AVGs are also characterized by their fast pace, high sensory-motor and cognitive load, and requirement for frequent, rapid motor responses to the presentation of multiple spatio-temporally unpredictable and fast-moving stimuli to ensure rapid switching between focused and distributed attentional states^6,17,18^. Therefore, not only do AVGs utilize many of the same visual attention skills reported to be impaired in dyslexia, they provide a highly rewarding experience that keeps the player engaged and motivated to improve their performance. Experienced AVG players reliably demonstrate faster magnocellular-temporal processing^19^, less activation in motion-sensitive regions (MT/MST) when viewing moving distractors, and less recruitment of the fronto-parietal attention network in response to increased attentional demands^20^. This suggests that AVG players more easily manage increased attentional demands and are better at suppressing distracting irrelevant information^20^. As such, AVG training for dyslexia may primarily act to improve magnocellular-dorsal stream efficiency^2^ and indirectly improve reading skills. However, to date, studies linking magnocellular and reading improvements following AVG training in dyslexia are limited^21^.

### AVG training in dyslexic children

A systematic review of the literature demonstrates AVGs to be a promising treatment for dyslexia^1^. The review found that AVGs are efficacious in improving reading rate and fluency, which has been shown to be the most stable and reliable index of dyslexia across both age and orthography (i.e., more than reading accuracy) in a recent meta-analysis^22^. The systematic review also reported that the few studies which examined reading accuracy or comprehension outcomes following AVG training, reported no improvement, suggesting more research into these reading subskills is required^1^. Furthermore, investigations are needed to identify the elements of dynamic attention that contribute most to the efficacy of AVG training, since AVGs may not all be equivalent. One option is to investigate whether efficacy could simply be enhanced by increasing the attentional demands via further reliance on eye movements that shift and direct attention^23^. Playing action games via eye tracking requires conscious motor direction of eye movements to make the appropriate motor actions needed to play, i.e., placing much greater demand on attentional flexibility and planning. Such volitional attention shifts are argued to be dependent on top-down signaling of prefrontal mechanisms (i.e., frontal eye fields and lateral prefrontal cortex), while signaling for automatic, bottom-up attention driven by stimulus properties originates from parietal regions^24,25^. Therefore, comparing regular AVG training, to AVG training with increased eye movement demands may enable dissociation of the parietal-fronto-attention network mechanisms most integral to attentional and reading improvements. While AVG studies to date have not assessed eye movement outcomes, other dynamic attention training programs have been shown to improve attention, reading and eye movements^26,27^ and so AVG studies are needed to build on these findings.

Of the ten published AVG training studies for dyslexia, most have used ‘Rayman Raving Rabbids’ with 12 h of training over 2 weeks^1–4,21,28–32^. Yet, preliminary evidence suggests that even a single AVG session may reduce reading errors immediately afterwards^30^, suggesting that shorter training may also be successful. However, this result must be interpreted with caution due to the small size of the study and the lack of a control group. Therefore, studies using different AVGs and shorter training times than used previously are important to extend the research base and determine optimal training length.

### The present study

The present study aimed to investigate whether AVG training, and in particular, AVG training with increased demands on dynamic visual attention via eye movements, would result in greater improvements as compared with a comparison group receiving only treatment-as-usual school-based reading intervention. Text reading accuracy, rate and comprehension, eye movement behaviour during rapid naming, and magnocellular tasks of temporal efficiency were included as outcome measures. ‘Fruit Ninja’^33^, a simple and non-violent fruit-slicing game that meets AVG criteria, was selected for use in both AVG training groups. It has not previously been investigated. A training duration of 5 h (ten, 30-min sessions over a 2-week period) was used to determine if a shorter duration than the 12 h used in most previous studies would also lead to improvement in reading. Those in the AVG training group with increased attentional demands (AVG +) played Fruit Ninja using eye tracking to control the cursor on the screen, while those in the regular AVG training group (AVG-R) played using a standard computer mouse, comparable to the motor controllers used for most video game consoles.

It was hypothesized that:

1. Dynamic attentional training, using AVGs, would lead to significantly greater improvement in reading rate and rapid naming than the treatment-as-usual only comparison group. The benefit to reading accuracy, comprehension, eye movements and magnocellular measures were also explored.
2. AVG+ training, with increased attention demands via eye movements, would be more effective than regular AVG training (i.e., AVG-R).

## Results

### Reading improvements

**Figure 1:**
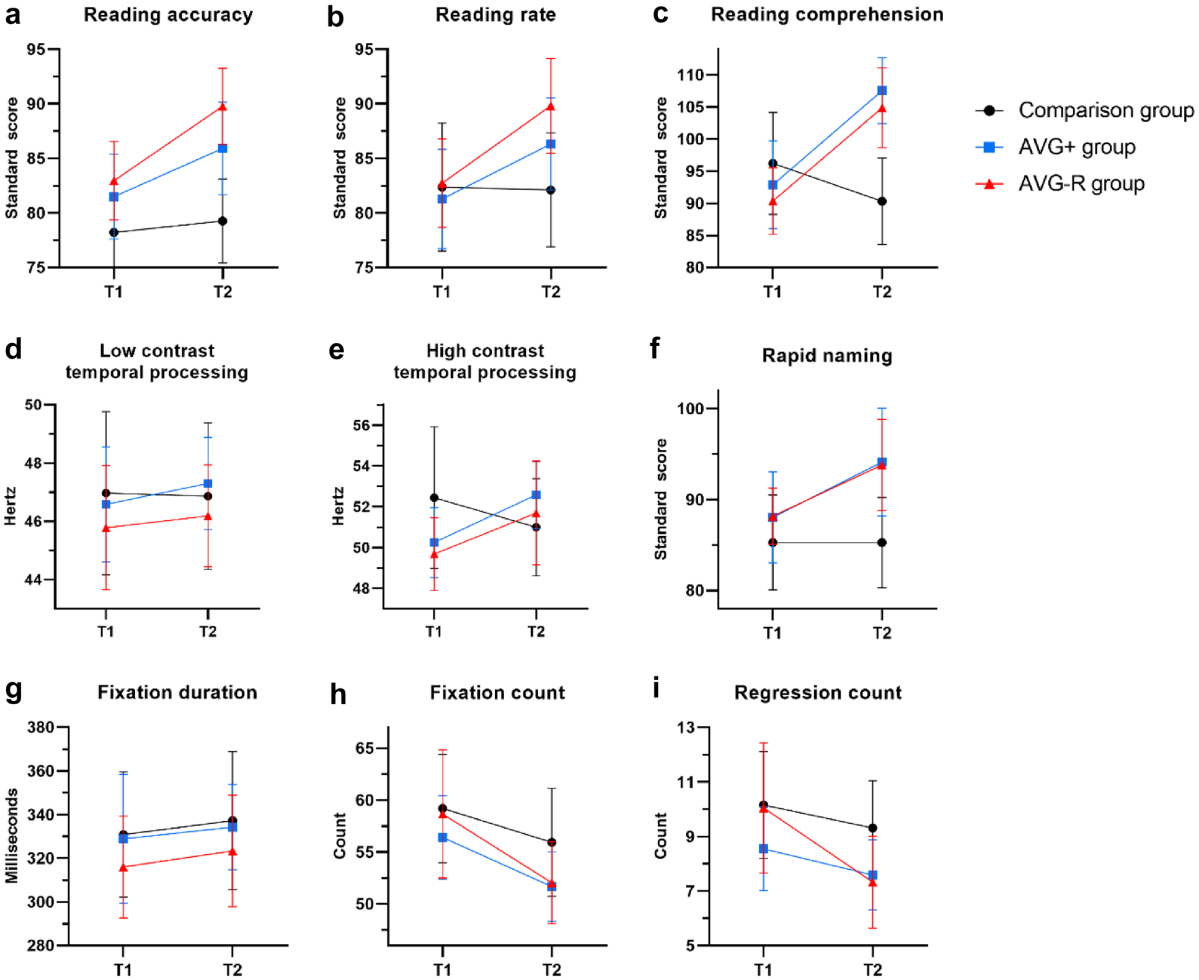
Dyslexic children’s performances before (T1) and after (T2) AVG+ training, AVG-R training or treatment-as-usual (comparison group) on (**a**) reading accuracy, (**b**) reading rate, (**c**) reading comprehension, (**d**) low contrast temporal processing, (**e**) high contrast temporal processing, (**f**) rapid naming, (**g**) fixation duration, (**h**) fixation count), and (**i**) regression count. Means and 95% confidence intervals are displayed.

**Figure 2:**
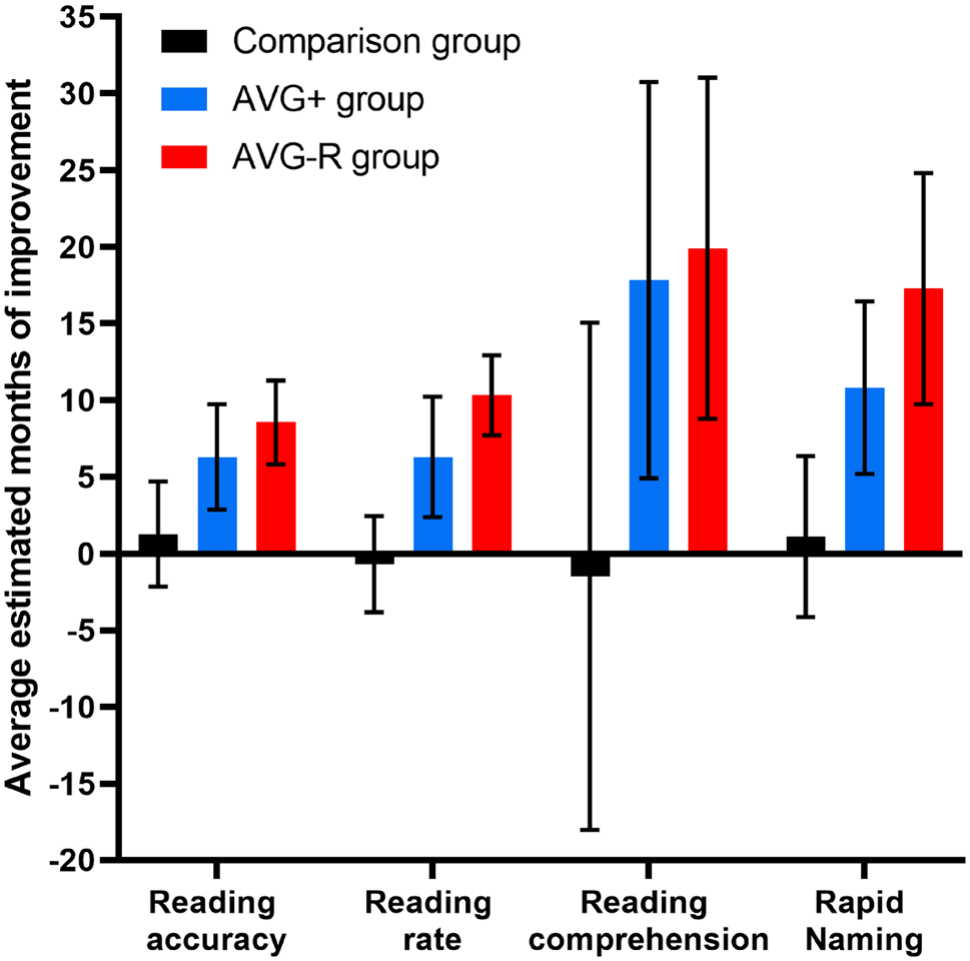
Dyslexic children’s average estimated months of improvement in performances on reading and rapid naming following AVG+ training, AVG-R training or treatment-as-usual (comparison group). Improvement estimates are based on the change between T1 and T2 in normative age equivalent estimates provided by the clinical test manuals (i.e., YARC, CTOPP-2). Means and 95% confidence intervals are displayed.

As shown in Table 1, a Two-Way Mixed Design analysis of variance (ANOVA) revealed a significant interaction effect between time and intervention for reading accuracy. Simple effects analysis showed significant differences between groups post-intervention (T2), but not at baseline (T1). Simple effects analysis followed by pairwise comparisons indicated that reading accuracy significantly improved only in the AVG groups between T1 and T2, with a comparable level of improvement in the AVG+ and AVG-R groups, *p* = .418 (Fig. 1). The average improvement in reading accuracy, as based on normative age equivalent estimates from the York Assessment of Reading for Comprehension—Primary Reading (YARC) assessment, was: AVG+  = 6.31 months, AVG-R = 8.55 months, and comparison group = 1.26 months (see Fig. 2). Descriptives and Standard Mean Differences (SMDs; Hedges *g*), are shown in Table 2.

**Table 1:** Analysis of variance results and summary of post hocs for the effects of intervention on of reading, eye movements and magnocellular tasks.

	Time × intervention	Simple effects for time		Simple effects for intervention
Reading accuracy	Sig. Wilk’s λ = .83, *F*(2, 60) = 6.00, *p* = .004, η_p_^2^ = 0.17	Comparison (T1 = T2) AVG+ (T1 < T2) AVG-R (T1 < T2)	T1: *p* = .160, η_p_^2^ = 0.06 T2: *p* = .001, η_p_^2^ = 0.21	T1: Comparison = AVG+ = AVG-R T2: Comparison ≠ AVG+ or AVG-R; AVG+ = AVG-R
Reading rate	Sig. Wilk’s λ = .74, *F*(2, 60) = 10.79, *p* < .001, η_p_^2^ = 0.27	Comparison (T1 = T2) AVG+ (T1 < T2) AVG-R (T1 < T2)	T1: *p* = .893, η_p_^2^ = 0.04 T2: *p* = .048, η_p_^2^ = 0.09	T1: Comparison = AVG+ = AVG-R T2: Comparison ≠ AVG+ or AVG-R; AVG+ = AVG-R
Reading comprehension	Sig. Wilk’s λ = .79, *F*(2, 60) = 7.91, *p* = .001, η_p_^2^ = 0.21	Comparison (T1 = T2) AVG+ (T1 < T2) AVG-R (T1 < T2)	T1: *p* = .444, η_p_^2^ = 0.03 T2: *p* < .001, η_p_^2^ = 0.25	T1: Comparison = AVG+ = AVG-R T2: Comparison ≠ AVG+ or AVG-R; AVG+ = AVG-R
Rapid naming	Sig. Wilk’s λ = .81, *F*(2, 60) = 6.95, *p* = .002, η_p_^2^ = 0.19	Comparison (T1 = T2) AVG+ (T1 < T2) AVG-R (T1 < T2)	T1: *p* = .622, η_p_^2^ = 0.02 T2: *p* = .035, η_p_^2^ = 0.11	T1: Comparison = AVG+ = AVG-R T2: Comparison ≠ AVG+ or AVG-R; AVG+ = AVG-R
Fixation duration	NS Wilk’s λ = .99, *F*(2, 60) = 0.03, *p* = .971, η_p_^2^ = 0.01	Comparison (T1 = T2) AVG+ (T1 = T2) AVG-R (T1 = T2)	T1: *p* = .668, η_p_^2^ = 0.01 T2: *p* = .727, η_p_^2^ = 0.01	T1: Comparison = AVG+ = AVG-R T2: Comparison = AVG+ = AVG-R
Fixation count	NS Wilk’s λ = .98, *F*(2, 60) = 0.45, *p* = .641, η_p_^2^ = 0.02	Comparison (T1 = T2) AVG+ (T1 > T2) AVG-R (T1 > T2)	T1: *p* = .816, η_p_^2^ = 0.01 T2: *p* = .184, η_p_^2^ = 0.06	T1: Comparison = AVG+** = **AVG-R T2: Comparison = AVG+ = AVG-R
Regression count	NS Wilk’s λ = .97, *F*(2, 60) = 0.81, *p* = .449, η_p_^2^ = 0.03	Comparison (T1 = T2) AVG+ (T1 = T2) AVG-R (T1 > T2)	T1: *p* = .539, η_p_^2^ = 0.02 T2: *p* = .092, η_p_^2^ = 0.08	T1: Comparison = AVG+ = AVG-R T2: Comparison = AVG+ = AVG-R
Flicker fusion 5%	NS Wilk’s λ = .99, F(2, 60) = .14, *p* = .872, η_p_^2^ = .01	Comparison (T1 = T2) AVG+ (T1 = T2) AVG-R (T1 = T2)	T1: *p* = .748, η_p_^2^ = 0.01 T2: *p* = .686, η_p_^2^ = 0.01	T1: Comparison = AVG+ = AVG-R T2: Comparison = AVG+ = AVG-R
Flicker fusion 75%	NS Wilk’s λ = .92, *F*(2, 60) = 2.78, *p* = .070, η_p_^2^ = .09	Comparison (T1 = T2) AVG+ (T1 < T2) AVG-R (T1 = T2)	T1: *p* = .251, η_p_^2^ = 0.05 T2: *p* = .564, η_p_^2^ = 0.02	T1: Comparison = AVG+ = AVG-R T2: Comparison = AVG+ = AVG-R

Sig = significant; NS = Non-significant; AVG-R = Action Video Game-Regular Group; AVG +  = Increased Attention Action Video Game Group; Comparison = Comparison Group.

**Table 2:** Averages, 95% confidence intervals and standard mean differences of outcome measures for each group and timepoint.

	AVG + group *n* = 23		AVG-R group *n* = 22		Comparison group *n* = 19		Standard mean differences (± CI)		
	T1 M (± CI)	T2 M (± CI)	T1 M (± CI)	T2 M (± CI)	T1 M (± CI)	T2 M (± CI)	AVG + versus comparison	AVG-R versus comparison	AVG + versus AVG-R
Reading accuracy	81.50 (3.68)	85.91 (4.01)	82.95 (3.39)	89.77 (3.30)	78.21 (2.90)	79.26 (3.60)	0.646 (0.63)	1.110 (0.66)	− 0.463 (0.60)
Reading rate	81.27 (4.30)	86.32 (3.98)	82.73 (3.83)	89.82 (4.09)	82.37 (5.48)	82.11 (4.87)	1.052 (0.65)	1.457 (0.69)	− 0.405 (0.59)
Reading comprehension	92.86 (6.42)	107.55 (4.82)	90.36 (4.89)	104.86 (5.85)	96.21 (7.42)	90.32 (6.29)	1.125 (0.66)	1.115 (0.66)	0.010 (0.59)
Rapid naming	88.04 (4.72)	94.13 (5.60)	87.85 (2.99)	93.81 (4.73)	85.26 (4.88)	85.26 (4.65)	1.059 (0.65)	1.038 (0.66)	0.024 (0.59)
Fixation duration	319.61 (21.88)	330.64 (17.81)	316.04 (21.84)	323.31 (24.04)	330.87 (26.80)	337.22 (29.45)	0.079 (0.62)	0.016 (0.62)	0.064 (0.61)
Fixation count	57.05 (3.75)	50.95 (2.95)	58.67 (5.79)	52.05 (3.69)	59.21 (4.87)	55.95 (4.84)	0.243 (0.62)	0.287 (0.62)	− 0.044 (0.60)
Regression count	8.81 (1.42)	7.24 (1.04)	10.05 (2.24)	7.33 (1.59)	10.16 (1.83)	9.32 (1.62)	0.159 (0.62)	0.409 (0.63)	− 0.249 (0.61)
Flicker fusion 5%	46.58 (1.87)	47.29 (1.50)	45.77 (2.11)	46.18 (1.65)	46.96 (2.61)	46.85 (2.34)	0.172 (0.61)	0.105 (0.62)	0.059 (0.60)
Flicker fusion 75%	50.25 (1.62)	52.58 (1.56)	49.89 (1.72)	51.91 (2.39)	52.44 (3.24)	50.99 (2.22)	0.705 (0.63)	0.604 (0.63)	0.101 (0.59)

AVG-R = Action Video Game-Regular Group; AVG+  = Increased Attention Action Video Game Group; ± CI =  + / − 95% Confidence Interval; Standard Mean Differences are interpreted as small = 0.2, moderate = 0.5 and large = 0.8, with positive scores in favour of the first group listed in the comparison.

A similar pattern of results was observed for reading rate and reading comprehension, which also showed a significant time and intervention interaction effect. For both measures, the three groups did not differ in performance at T1. Again, the AVG groups, but not the comparison group, had improved significantly at T2, with the improvement in both reading rate and reading comprehension comparable between the AVG+ and AVG-R groups (*p* = .754 and *p* = .999, respectively). The average improvement in reading rate was equivalent to: AVG+ group = 6.31 month, AVG-R group = 10.33 months, and comparison group = − 0.69 months (see Fig. 2). The average improvement in reading comprehension was equivalent to: AVG+ = 17.82 months, AVG-R = 19.90 months, and comparison group = − 1.48 months.

### Rapid automatic naming and eye movements

As illustrated in Fig. 1, ANOVA showed a signification time and intervention interaction effect for rapid naming (see Table 1 for analysis results and Table 2 for descriptives and SMDs). No group differences were observed at T1, while only the AVG groups showed significantly improved naming speed at T2, with no difference in performance occurring for the comparison group between T1 and T2. There was no difference in amount of improvement between the AVG+ and AVG-R groups at T2, *p* = .999. The average improvement in rapid naming, as based on normative age equivalent estimates from the Comprehensive Test of Phonological Processing, 2nd Edition (CTOPP-2), was equivalent to: AVG+ = 10.82 months, AVG-R = 17.28 months, and comparison group = 1.10 months (see Fig. 2).

No significant effects were observed for fixation durations during rapid naming, as no change in the duration of fixations was observed between groups at either T1 or T2 for this task. For fixation count, the interaction effect between time and intervention, and the main effect of intervention group were not significant. However, the main effect for time was significant, indicating a general reduction in number of fixations between T1 and T2 for rapid naming. Similar results were obtained for regression count, where only a significant main effect for time was observed, again indicating a general reduction in regressive saccades during rapid naming between T1 and T2 (see Fig. 1).

### Magnocellular temporal processing

ANOVAs revealed no significant interaction or main effects for low (5%) or high (75%) contrast flicker fusion tasks, indicating no significant changes in detection thresholds between groups from baseline to post-intervention (Fig. 1); however, there were moderate effect sizes for both the AVG+ and AVG-R groups as compared with the comparison group (Table 2). Inspection of the confidence intervals also indicated a high degree of variability of Flicker Fusion Threshold (FFT) performance at both T1 and T2.

Due to the high variability in FFT performance, further investigation was performed to examine whether there was an underlying association between the following:

1. initial flicker fusion ability (i.e., baseline performances at T1) and reading outcomes following the training period, and;
2. amount of improvement in flicker performance and reading outcomes following the training period.

Improvements in the outcome measures were calculated as post-training score (T2) minus baseline (T1) score, and all participants were analysed as a single group to increase power.

Pearson correlational analyses indicated that flicker fusion performance at baseline (T1) significantly and negatively correlated with improvements in temporal processing following the training period, indicating that lower initial flicker performances were associated with greater FFT improvement following training. Low contrast flicker fusion at baseline also correlated significantly and negatively with reading accuracy improvements after the training period, showing that lower initial low contrast flicker performance was associated with greater reading accuracy improvement following training. Additionally, following training, the amount of improvement in low contrast flicker fusion was significantly and positively correlated with reading accuracy improvements, suggesting that those who experienced the most improvement in low contrast FFT after the training period also experienced greater reading accuracy improvements (See Table 3). Results of exploratory correlational analyses, with only the AVG participants included, were similar and are provided in the supplementary information document.

**Table 3:** Correlations between flicker fusion performance and reading improvement scores.

	Baseline 5% FFT (T1)	Baseline 75% FFT (T1)	Reading accuracy improvement	Reading rate improvement	Reading comp improvement	Rapid naming improvement	5% FFT improvement	75% FFT improvement
Baseline 5% FFT (T1)	–	.593**	− .250*	− .049	.006	.008	− .650**	− .171
Baseline 75% FFT (T1)		–	− .208	− .102	− .036	.111	− .294*	− .617**
Reading accuracy Improvement			–	.345**	.233	− .180	.357**	.089
Reading rate improvement				–	.385**	− .285*	− .065	.122
Reading comp improvement					–	− .390**	− .092	.169
Rapid naming improvement						–	− .037	− .186
5% FFT improvement							–	.200
75% FFT improvement								–

According to Cohen’s guidelines, *r* ≥ 0.10, *r* ≥ 0.30, and *r* ≥ 0.50, represent small, medium, and large effect sizes, respectively; Improvements scores were calculated as post-training score (T2) minus baseline (T1) score.

*FFT* Flicker Fusion Threshold (Hz).

*p < .05, ** *p* < .01.

Based on the significant correlations shown in Table 3, regression analyses were conducted to assess the contribution of low contrast flicker fusion performance at baseline (T1) to improvements in temporal processing following the training period, and to assess the contribution of post-training period improvements in temporal processing to degree of improvements in reading accuracy. All participants (*N* = 64) were included in these analyses. Lower low contrast flicker fusion scores at baseline significantly predicted greater improvement in low contrast flicker fusion performance following the training period, explaining 42.2% of the variance in the regression model; *F* (1, 61) = 44.560, *β* = − 0.650, *p* < .001. Improvement in low contrast flicker fusion following the training period was then found to be a significant predictor of improvement in reading accuracy following training, explaining 12.7% of the variance in the regression model; *F* (1, 60) = 8.754, *β* = 0.357, *p* = .004. An exploratory regression, which included only AVG participants, produced similar results and are provided in the supplementary information document.

## Discussion

The present study is the first to show that AVG training results in greater improvements in both text reading accuracy and comprehension as compared with a comparison group receiving only treatment-as-usual school-based reading intervention. We provide novel evidence that the greatest improvement in reading accuracy following training was found in participants who also showed the highest gains in low contrast magnocellular-temporal processing. Moreover, those with less proficient (i.e., lower) flicker fusion scores before training demonstrated the greatest improvement in temporal processing after training. The current study also investigated, for the first time, whether increased demands on dynamic visual attention via eye movements during AVG training would enhance training efficacy. While the use of a novel AVG with shorter training duration demonstrated efficacy comparable to past research, AVG efficacy was not enhanced by increasing the demand on dynamic attention using eye movements. This research contributes to the growing evidence demonstrating that dynamic attentional training using AVGs is an effective intervention for dyslexia.

Children who received AVG training (i.e., AVG+ or AVG-R) significantly improved in text reading accuracy, rate and comprehension, and rapid naming performance as compared with the comparison group, who did not show improvements. Yet, at T2 all three groups demonstrated fewer fixation and regression counts and unchanged fixation durations during rapid naming, suggesting that the increase in rapid naming score after AVG training may not be mediated by an increased efficiency of eye movements. However, inspection of the effect sizes suggests that there may be some reduction, albeit non-significant, in the number of forward and regressive fixations made by AVG participants. This may possibly reflect an improved capacity to shift between focused and distributed attention, as utilized by AVGs, and hence greater parafoveal processing capacity^34^ or a more generally increased ability to disengage from irrelevant stimuli. Alternatively, improved rapid naming following AVG training could indicate that as AVG participants have begun to experience increased success with game play and improved self-esteem in an educational setting, they would be less anxious and more confident and motivated when performing reading related tasks, such as rapid naming. Both groups of AVG training (AVG+ and AVG-R) using ‘Fruit Ninja’ resulted in at least 6 months of improvement across reading skills and rapid naming, with mostly large effect sizes (SMDs) found. The benefit to reading rate is comparable to studies using ‘Rayman Raving Rabbids’ for 12 h of training^1^. It is not clear whether the similar efficacy of the current results, despite only 5 h of training, may be driven by differences in the effectiveness of the AVGs used, or whether a plateau of intervention efficacy may start to occur after 5 h of training. This warrants further research.

The current findings also provide *novel* evidence showing that reading accuracy improvements following AVG training are related to gains in the temporal processing rate of the magnocellular stream at low contrast. Not only did AVG training result in improved reading accuracy, but the greatest degree of reading accuracy improvement was found in participants whose low contrast magnocellular-temporal processing also improved most after training. As all three groups demonstrated equivalent flicker fusion scores at baseline (T1), and the comparison group did not significantly improve in reading outcomes at T2, these changes must be attributed to the effect of AVG training. Improved magnocellular performance has previously been reported following other types of dynamic attention training for dyslexia^21,35,36^ and together all these findings are consistent with suggestions that the magnocellular stream activates the attention needed for early word recognition^37^. Impairments in these initial steps are theorized to cause a bottleneck that impacts later cognitive processes needed for word recognition—for example, orthographic-to-phonological mapping^37^.

As alluded to earlier, this study is the first to show that AVG training benefits text reading comprehension. The benefit is likely to be secondary to improvements in reading accuracy and rate. With less cognitive effort required for these primary skills, readers can focus their cognitive and attentional capacity more on comprehension. Improvements in skills that underpin the comprehension process, such as working memory and executive functioning), may also explain the improved comprehension demonstrated here, as these skills have also been shown to benefit from AVGs^6^. Either way, further research is needed to confirm these proposals.

Furthermore, children with lower, less proficient flicker fusion scores at low contrast before training were more likely to show the greatest improvements in reading accuracy and in low contrast temporal processing following training, with post-training improvement in low contrast flicker fusion then predicting reading accuracy improvements. We conclude from this that AVG training may be most beneficial for dyslexic children with slower temporal processing, which has recently been identified to specifically characterise a subgroup of dyslexic children^38^.

In contrast to predictions, AVG+ training with increased dynamic attention demands via eye movements did not significantly mediate training efficacy. Those receiving AVG+ and AVG-R training improved comparably, though for most outcomes, AVG-R training tended to show larger, albeit nonsignificant, gains. This result may suggest that AVG efficacy as a reading intervention is more related to automatic attentional control rather than conscious attention and eye-movements. The other possible explanation is that placing a continued and increased demand on attention via eye movements may have inadvertently made game play more effortful and challenging, as evidence suggests that game difficulty should be adjusted commensurate with the players ability to maintain engagement^39^. Have also demonstrated that those who get better at playing AVGs over the course of training demonstrate the most cognitive gains. While game scores were not formally monitored in the current study, those in the AVG+ group scored consistently lower than the AVG-R group throughout training. Therefore, it is possible that the AVG+ version of training required much greater neural resources resulting in a higher level of difficulty for children to play and hence occasioned greater cognitive fatigue. The practical advantage of the findings of the current study is that AVG-R training can more easily be implemented in a range of settings without the need for specialist eye tracking equipment or training.

Consideration must also be given to the other key driver of learning and neural plasticity that AVGs include—i.e., both intrinsic and extrinsic reward-driven motivated behaviour^16^—as well as its interplay with attentional control. AVGs clearly engage emotional processes, which also plays a role in attentional control. For example, the magnocellular pathway, considered the most common neurobiological explanation for the visual attentional impairments seen in dyslexia^14,40,41^, includes subcortical projections to the emotional attention processing amygdala^42,43^. As such, it will be important for future studies to examine these processes in order to elucidate the mechanisms underlying AVGs efficacy as a reading intervention.

In conclusion, dynamic attentional training using the AVG, ‘Fruit Ninja’, for as little as 5 h significantly improves reading accuracy, rate, comprehension and rapid naming in dyslexic children, despite not directly training reading. Participants whose low contrast magnocellular-temporal processing improved most following training also showed significantly greater improvement in text reading accuracy. The short training duration, however, did not result in significant improvements to eye movements. Increasing attentional demands by increasing reliance on conscious top-down direction of eye movements during game play also did not increase efficacy, rather the results suggest it may have been cognitively fatiguing. Nonetheless, the current evidence supports the view that dynamic visual attention plays an integral role in dyslexia and reading. The study also highlights the clinical applicability of AVGs as a fun, engaging intervention for reading that can improve aspects of reading that are not generally improved with current phonics treatments. AVG training is less resource-demanding than current options^2,44^ and could easily be implemented as a reading intervention in a variety of settings, including schools. Further research is needed to continue investigation into the dynamic attentional mechanisms that drive AVG efficacy, assess longer-term follow up of outcomes, and directly compare AVG and phonics-based interventions. Future investigations should also consider the role of motivational engagement in the efficacy of AVG games.

## Methods

### Participants

A total of 64 dyslexic children aged 8;09 to 13;01 years (Grades 3–6) were recruited from Melbourne metropolitan primary schools to participate in the study. Potential participants were first selected on the basis of the school indicating they had a reading problem or had an existing formal diagnosis of dyslexia. They were then assessed by the research team to determine whether they met inclusion criteria. Confirmation of dyslexia was made in accordance with DSM-5^45^ criteria for a Specific Learning Disorder in Reading, which is currently the most common diagnostic criteria used by psychologists. More specifically, in order to be included in the study, participants required (1) a history of reading difficulties as reported by teachers or parents and/or a formal diagnosis of dyslexia, AND (2) current reading performance at least 1 SD^45,46^ below age-standardized norms in one or more area of reading (text reading accuracy, rate and/or comprehension) on the YARC^47^. This mitigated the risk of false positives since confirmation of dyslexia was not simply based on a single timepoint of testing data, but was based on converging evidence, with diagnoses confirmed by a psychologist on the research team. Participants were also required to have normal intelligence (Standard score ≥ 85 for age on the Raven’s Matrices test), normal or corrected-to-normal vision and hearing, and English as their primary language. Children with known medical and neurodevelopmental disorders other than dyslexia, or who did not meet the study criteria for dyslexia, were excluded (*N* = 16).

Parents/guardians of participants provided written informed consent for their child to engage in the study and all children who participated provided verbal assent. Parents/Guardians of participants also completed a questionnaire about their child’s video game usage. While all participants engaged in video games at the time of the study, approximately half of participants regularly played a range of video game types that included AVGs, while the other half played only non-AVGs. A similar pattern of game usage was seen across the intervention and comparison groups. At the commencement of training, participants were asked to not play Fruit Ninja at home throughout the study duration. The participants were blind to the aims of the study. The study was registered as a clinical trial with the Australian New Zealand Clinical Trails Registry (registration number ACTRN12618001709235; registration dated 16/10/2018) and performed in accordance with the World Medical Association Declaration of Helsinki and with ethics approval granted by the La Trobe University Faculty Human Ethics Committee and the Victorian State Department of Education.

Participants at each school were randomly allocated using a random number generator to either AVG+ training (*n* = 23), AVG-R training (*n* = 22), or a ‘treatment-as-usual’ comparison group (*n* = 19). The comparison group were not provided with training by the researchers but continued to receive school-based reading remediation based on phonics-based programs, including ‘Jolly Phonics’ and ‘Reading Eggs’, as did all AVG players. ‘Jolly Phonics’ is a synthetic phonic program that teaches letter sounds, letter formation, blending, segmenting and irregular words. ‘Reading Eggs’ is a digital program that includes interactive videos and teaches phonics progressively, including phonemic awareness and phonics, letter-sound correspondence, blending and segmenting, letter identification, high-frequency words, vocabulary, sentence construction and comprehension. As shown in Table 4, groups did not significantly differ in chronological age or nonverbal intelligence. Groups also did not differ on reading accuracy (*p* = .160), reading rate (*p* = .893), reading comprehension (*p* = .444), or rapid number naming performance (*p* = .583) at baseline (T1; see Table 1 for descriptives), and were an average of 2.15 years behind age expectations in reading accuracy, 1.98 years behind in reading rate, and 0.97 years behind in reading comprehension.

**Table 4:** Baseline comparisons for age and non-verbal intelligence for intervention and comparison groups.

	AVG+ group *n* = 23	AVG-R group *n* = 22	Comparison group *n* = 19	*F* (2, 61)	*p*	η^2^
	*M* (*SD*)	*M* (*SD*)	*M* (*SD*)			
Age	10.37 (0.95)	10.49 (1.05)	10.73 (0.96)	0.695	.503	.02
Nonverbal intelligence	101.96 (8.75)	103.27 (9.09)	105.26 (7.68)	0.777	.464	.02

### AVG training procedure

Children in each of the AVG groups completed dynamic attention training using AVGs in small groups of 3–4 in a quiet room at their school. The ten, 30-min sessions occurred each weekday for two weeks, for a total of 5 h. Both AVG training groups played Fruit Ninja via the android emulator, BlueStacks App Player^48^, on a 23 inch Dell computer screen to minimise any differences between training methods (e.g., screen size) as the eye tracking program required a Windows operating system.

‘Fruit Ninja’ meets AVG criteria as it requires players to quickly slice multiple fruit that move rapidly with temporal and spatial unpredictability from the periphery of the screen, with points awarded for each fruit sliced. Players must also switch between following a target and monitoring the entire scene as well as planning and inhibiting responses so that non-targets (i.e., ‘bombs’) are avoided, and must rapidly make decisions about how best to respond to the visual scene to achieve the most points.

The main aim of ‘Fruit Ninja’ is to slice as many fruits as possible. Players must make a single swipe motion through each fruit to earn a point, with extra points awarded for slicing multiple fruits with one swipe (called combos) or slicing ‘special’ fruit. Children in both AVG training groups were allowed to freely play any of the Fruit Ninja mini-games during their training sessions. Scores in each mini-game earn game currency and increase the players experience points, helping players progress to the next level and gain access to new features. Players then use game currency to buy items that provide additional powers for use during the games. Children could also complete various missions (e.g., slice 8 green apples in one game) to earn additional game currency.

#### AVG-R training

Children in the AVG-R group played Fruit Ninja using a computer mouse to control the cursor on the screen. They were required to move the mouse in a slicing motion while holding down the left button in order to slice fruit.

#### AVG+ training

Children in the AVG+ group played Fruit Ninja by using their eye movements to control the cursor on the screen. This was theorized to place an increased demand on dynamic visual attention through accurate and well-timed eye movements. During training sessions, participants had their eye movements tracked binocularly using a Gazepoint GP3HD screen mounted infrared camera with 150 Hz sampling rate. The Gazepoint Fruit Ninja application programming interface was also used to translate eye movements into cursor movement during AVG play. Before each training session, participants would undergo a 9-point calibration procedure. Participants were provided with a chin and forehead rest to reduce movement for initial training sessions, and as needed for later training sessions (i.e., if head movement resulted in eye tracker drop out), though almost all children in the AVG+ group adapted sufficiently and quickly in keeping their head still while just moving their eyes.

### Materials

All participants completed cognitive and reading assessment 3 to 5 days before (Baseline; T1) and after (T2) the training period (i.e., a total of 20–24 days apart) with tasks administered in randomized order. Assessments occurred individually in a quiet room at the child’s school. Participants completed all computerised and psychophysical tasks, including AVG training, at a viewing distance of about 59 cm.

#### Nonverbal intelligence

Nonverbal intelligence was assessed at baseline using the Ravens Coloured Progressive Matrices for participants aged 5–11 years or the Ravens Standard Progressive Matrices for participants aged 12 + years^49^. Each test contains a series of matrices of increasing complexity. Age-based standard scores were calculated using normative data.

#### Text reading

The YARC was used to assess text reading accuracy, rate and comprehension skills^47^ . The task requires children to successfully read two passages of text aloud, while being timed, and answer questions about each text to assess both literal and inferential text comprehension. The two passages to be read are selected from a series of seven passages of increasing difficulty, corresponding to each grade level of primary school. Passage selection is based on each child’s grade level and reading proficiency in accordance with the YARC manual. Equivalent passage levels from alternative forms (A and B) were used for T1 and T2, in a counterbalanced order. Age-based standardized scores and age equivalence estimates for reading accuracy, rate and comprehension performances were used. FastaReada^50^, a psychophysical measure of reading fluency, was also included in data collection, but majority of participants were not able to reliably pass the practice trial, and so the task has been excluded from data analysis (See supplementary document).

#### Rapid automatic naming

The number rapid naming task from the CTOPP-2 was assessed at both T1 and T2. The task, a strong predictor of reading, measures rate of visual to verbal information processing. It was also used to study changes in eye movement behaviour as it minimizes stimulus-based factors known to influence eye movements, including word difficulty, length and predictability^12^. Participants were required to rapidly name aloud 36 stimuli (four lines of nine stimuli). Time taken to name all stimuli was recorded and standardized scores are reported. The letter RAN task from the CTOPP-2 was also completed by participants, but as eye movement results between the number and letter versions were comparable, results have not been included further for brevity (See supplementary document).

#### Eye movements during rapid automatic naming

Eye movements were recorded binocularly during the rapid naming task using a Gazepoint GP3HD screen mounted infrared camera with 150 Hz sampling rate. The GP3HD tracks vertical and horizontal eye positions with an average gaze position accuracy of 0.5 degrees. Participants had their head placed in a chin and forehead rest to reduce movement. Before beginning the task, each participant underwent a 9-point eye movement calibration procedure. The variables, fixation duration, fixation count, and regression count were extracted for statistical analysis. Fixation duration was calculated as the average (mean) temporal length of fixations, fixation count was defined at the total number of fixations made, and regression count was defined as the number of backward saccades made across previously named stimuli.

#### Magnocellular temporal processing tasks

As it is theorized that AVGs improve reading via the magnocellular system, two achromatic flicker fusion tasks modulated at high (75%) and low (5%) luminous contrast were included as surrogate measures of the temporal processing thresholds of the magnocellular pathway previously. The tasks were previously used by Brown and colleagues^51^, and were assessed at T1 and T2. Four LEDs conveyed light into separate 6 mm diameter fibre optic light guides which were presented flush in a free-standing wooden panel in a diamond-array subtending 1.0◦, center-to-center, at the eye. Each task consisted of a four-alternative forced-choice design with 32 trials and used a Parameter Estimation by Sequential Testing (PEST) procedure (For further details about task design, see ^51^). Participants were instructed that one LED light per trial (demarcated by a high-pitched beep) would flicker for 3 s and at the end of the trial (indicated by a low-pitched beep) they were required to indicate which light source they saw flicker or guess when they were unsure. The order of high and low contrast conditions was counterbalanced to control for practice effects, and participants were provided with a familiarization practice session. Participants completed the tasks in a dimly lit room. The start of each trial was manually controlled by the experimenter to ensure participants were looking at the display, ready for the trial to commence.

### Data analysis

An a priori power analysis indicated that there was 95% power to detect a large effect size at *p* = .05 with 18 participants per group. As adjustment or removal of outliers represents a potential source of bias in intervention trials, handling of outliers was conducted in accordance with the Statistical Principles for Clinical Trials Guidelines (1998). Several outliers just outside the normal distribution were identified, but not found to influence results, so were retained (i.e., no observations were excluded). Standard scores, rather than raw scores, for clinical tasks were used to analyse performance change between T1 and T2 as they capture meaningful changes in performance as based on age-normative data. For normally distributed variables, the SMD (Hedges *g*), an effect size measure comparing the changes (T2-T1) between two groups, was calculated for each outcome variable to compare the efficacy of each AVG group to the comparison group, and compare the efficacy of the AVG+ and AVG-R groups to each other. The magnitude of SMD is interpreted as small = 0.2, moderate = 0.5 and large = 0.8^52^. Positive SMDs are in favour of the first group listed within the comparison. Normality was confirmed via assessment of skewness and kurtosis, Kolmogorov–Smirnov values, and visual inspection of histograms and box plots.

To determine whether the AVG groups improved significantly more than the comparison group, two-way mixed design (time [T1 and T2] by group [AVG+, AVG-R, comparison group]) ANOVAs were conducted for each outcome. Pairwise comparisons of outcomes between groups at T1 and T2 were then used to determine whether the AVG+ group showed greater improvement to the AVG-R group. Means and confidence intervals for each outcome variable, group and time point is shown in Table 2. Correlation and regression analyses were then used to explore the relationship between flicker fusion performances and improvements in reading outcomes following AVG training.

To assist with interpretation of results in clinically meaningful terms, normative age equivalent estimates from the clinical test manuals (i.e., YARC, CTOPP-2) were used to provide an estimate of average months of improvement.

## Supplementary Information

Supplementary Information.

## Data Availability

The study was registered as a clinical trial with the Australian New Zealand Clinical Trails Registry, http://www.anzctr.org.au/Trial/Registration/TrialReview.aspx?id=376081 (registration number ACTRN12618001709235; registration dated 16/10/2018). The data have not been made available on a permanent third-party archive, but requests for the data can be sent via email to the lead author.
