# Supplementary material for: Action video game training improves text reading accuracy, rate and comprehension in children with dyslexia: a randomized controlled trial
Source: Sci Rep. 2021 Sep 20;11:18584. doi: 10.1038/s41598-021-98146-x (PMC8452648; doi:10.1038/s41598-021-98146-x)
Supplement: Supplementary file 1 — Supplementary Information. [file 41598_2021_98146_MOESM1_ESM.docx]

Supplementary Information

Action Video Game Training improves Text Reading Accuracy, Rate and Comprehension in Children with Dyslexia: A Randomized Controlled Trial

Jessica L. Peters^1*^, Sheila G. Crewther^1^, Melanie J. Murphy^1^, & Edith L. Bavin^1,2^.

^1^Department of Psychology and Counselling, La Trobe University, Melbourne, Australia;

^2^Intergenerational Health, Murdoch Childrens Research Institute, Melbourne, Australia.

*Corresponding Author:

Jessica Peters

E: [j.peters@latrobe.edu.au](mailto:j.peters@latrobe.edu.au)

Department of Psychology and Counselling,

La Trobe University, Melbourne, Victoria 3086, Australia

Supplementary Materials

FastaReada

FastaReada is a computerized measure of reading fluency. Children are presented with six words from a passage of text during each trial and are asked to read the six words aloud as accurately as possible. The presentation time of each trial is increased each time all six words are read aloud correctly, or decreased if errors are made, via a PEST adaptive staircase algorithm based on a maximum-likelihood threshold estimation. Before commencing the task, children are told that the presentation duration may before so short that they may not be able to read all six words, but were encouraged to attempt each trial nonetheless.

Inspection Time

A computerised Inspection Time task aimed to assess visual processing by determining the fastest exposure time required to reliability identify simple stimuli (A fish, truck or butterfly; task images shown below). For each trial, the child was instructed to identify which stimulus was shown, or guess if they were unsure. A PEST procedure with a six reversal threshold was used to determine the exposure determination of subsequent trials. Performance was determined by the fastest exposure time at which the child could reliably identify the stimulus.


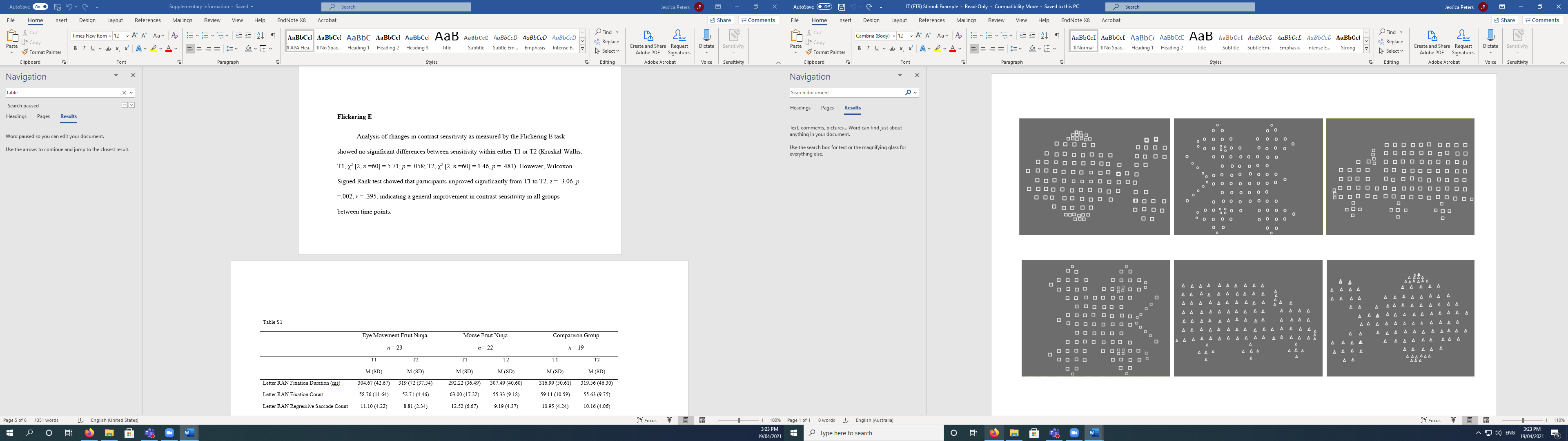


Flickering E

A flicker-defined form contrast sensitivity task (referred to as ‘Flickering E’) developed for Barnard, Crewther, and Crewther (1998) and used by Laycock, Crewther, and Crewther (2012) among others, was completed at T1 and T2. Stimuli consisted of the letter E, subtending 5 degrees, in four orientations (north-east, south-east, south-west, north-west) and of variable contrast presented at fixation. The ‘E’ form was created by a distribution of light and dark dots on a mid-gray background creating an illusory edge. The foreground and background alternated between dark and light at a rate of 58.5 Hz. The task consisted of a four-alternative forced-choice design and a PEST procedure to provide an estimate of contrast threshold, terminating once the confidence level exceeded 90% (for further details about task design, see Laycock et al., 2012). Participants were instructed that one stimulus would appear per trial and at the end of the trial they were required to indicate which stimulus orientation they saw or guess when unsure.

Supplementary Results

Reading Fluency (FastaReada)

It was decided that the FastaReada task was not an appropriate measure of reading fluency for the dyslexic population used in the current study as 29% of participants were not able to complete the task (performances were at task floor). Nonetheless, results for the 45 participants (EMAVG *n* = 15; MouseAVG *n* = 18; Comparison Group *n* = 12) who were able to complete the task are provided but should be interpreted with caution.

Wilcoxon Signed Rank test indicated that participants improved from T1 to T2, *z* = -2.14, *p* =.032, *r* = .319. Kruskal-Wallis tests were conducted to between group differences in reading fluency performance for each timepoint (T1 and T2) revealed that groups did not significantly differ in reading fluency (FastaReada) performance at T1, χ^2^ (2, *n* =45) = 0.94, *p* = .626, or at T2, χ^2^ (2, *n* =45) = 4.41, *p* = .110, indicating that all groups showed improved reading fluency over time.

## Improvements to Eye Movements during Rapid Letter Naming

Letter RAN Fixation Duration

There was no significant interaction effect between time and intervention, Wilk’s Lambda = .99, *F*(2, 60) = 0.260, *p* =.699, partial eta squared = .012. The main effect of time, Wilk’s Lambda = .96, *F*(1, 60) = 2.54, *p* =.116, partial eta squared = .042, and the main effect for intervention group were also not significant, *F*(2, 60) = 1.519, *p* =.227, partial eta squared = .050, indicating that no group showed significantly improved fixation duration during rapid naming of letters at T2. The SMDs comparing each AVG group and the comparison group were small, EMAVG SMD = 0.229, MouseAVG SMD = 0.229, while the SMD comparing the EMAVG and MouseAVG groups was negligible, SMD = 0.000.

Letter RAN Fixation Count

There was no significant time*intervention interaction effect for fixation count, Wilk’s Lambda = .98, *F*(2, 60) = 0.48, *p* =.623, partial eta squared = .016. The main effect of time was significant, Wilk’s Lambda = .84, *F*(1, 60) = 10.75, *p* =.002, partial eta squared = .156, however, the main effect for intervention group was not significant, *F*(2, 60) = 0.79, *p* =.459, partial eta squared = .027, indicating that all groups showed similarly reduced fixation counts during rapid naming of letters at T2. The SMDs comparing each AVG group and the comparison group were small, EMAVG SMD = 0.194, MouseAVG SMD = 0.316, while the SMD comparing the EMAVG and MouseAVG groups was negligible, SMD = -0.122.

Letter RAN Regressive Saccade Count

There was no significant interaction effect between time and intervention, Wilk’s Lambda = .96, *F*(2, 60) = 1.16, *p* =.321, partial eta squared = .038. The main effect of time was significant, Wilk’s Lambda = .85, *F*(1, 60) = 9.91, *p* =.003, partial eta squared = .146. The main effect for intervention group was not significant, *F*(2, 60) = 0.33, *p* =.717, partial eta squared = .011, indicating that all groups showed a similar decrease in regressive saccades during rapid naming of letters between T1 and T2. The SMDs comparing each AVG group and the comparison group were small to moderate, EMAVG SMD = 0.290, MouseAVG SMD = 0.493, while the SMD comparing the EMAVG and MouseAVG groups was small, SMD = -0.203.

Inspection Time – Visual Processing Speed

The inspection time task was used to assess the effect of intervention on visual processing speed. There was no significant interaction effect between time and intervention, Wilk’s Lambda = .98, F(2, 61) = 0.45, p =.642, partial eta squared = .014. The main effect of time, Wilk’s Lambda = .80, F(1, 61) = 15.17, p >.001, partial eta squared = .199, and the main effect for intervention group, F(2, 61) = 7.72, p =.001, partial eta squared = .202, were both significant. Pairwise comparisons showed that only the AVG groups improved significantly between T1 and T2; EMAVG, mean difference = 10.56, p =.004; MouseAVG, mean difference = 8.48, p =.021; comparison group, mean difference = 5.63, p =.150. The SMDs between each AVG group and the comparison group were small, EMAVG SMD = 0.278, MouseAVG SMD = 0.113. Pairwise comparisons indicated that the EMAVG and MouseAVG groups improved comparably, p =.999, SMD = 0.162.

Flickering E

Analysis of changes in contrast sensitivity as measured by the Flickering E task showed no significant differences between sensitivity within either T1 or T2 (Kruskal-Wallis: T1, χ^2^ [2, *n* =60] = 5.71, *p* = .058; T2, χ^2^ [2, *n* =60] = 1.46, *p* = .483). However, Wilcoxon Signed Rank test showed that participants improved significantly from T1 to T2, *z* = -3.06, *p* =.002, *r* = .395, indicating a general improvement in contrast sensitivity in all groups between time points.

| Table S1 |  | |  |  | |  |  | |
| --- | --- | --- | --- | --- | --- | --- | --- | --- |
|  | Eye Movement Fruit Ninja  *n* = 23 | |  | Mouse Fruit Ninja  *n* = 22 | |  | Comparison Group  *n* = 19 | |
|  |  |  |  |  |  |  |  |  |
|  | T1  M (SD) | T2  M (SD) |  | T1  M (SD) | T2  M (SD) |  | T1  M (SD) | T2  M (SD) |
| Letter RAN Fixation Duration (ms) | 304.67 (42.67) | 319 (72 (37.54) |  | 292.22 (36.49) | 307.49 (40.60) |  | 316.99 (50.61) | 319.56 (46.30) |
| Letter RAN Fixation Count | 58.76 (11.64) | 52.71 (4.46) |  | 63.00 (17.22) | 55.33 (9.18) |  | 59.11 (10.59) | 55.63 (9.75) |
| Letter RAN Regressive Saccade Count | 11.10 (4.22) | 8.81 (2.34) |  | 12.52 (6.67) | 9.19 (4.37) |  | 10.95 (4.24) | 10.16 (4.06) |
| Inspection time (ms) | 45.29 (17.57) | 34.74 (8.69) |  | 43.81 (15.94) | 35.33 (11.83) |  | 56.61 (17.09) | 50.98 (20.20) |
|  | T1  Median (IQR) | T2  Median (IQR) |  | T1  Median (IQR) | T2  Median (IQR) |  | T1  Median (IQR) | T2  Median (IQR) |
| FastaReada (Reading Fluency) | 189.00  (109.00-225.00) | 200.00  (155.25-218.75) |  | 184.50  (169.25-203.00) | 225.00  (180.00-437.00) |  | 184.50  (173.25-257.00) | 200.00  (182.25-209.00) |
| Flickering E (Contrast %) | .048  (.041 - .057) | .042  (.036 - .046) |  | .043  (.036 - .050) | .038  (.033 - .043) |  | .054  (.043 - .062) | .042  (.036 - .051) |

##

## Magnocellular Temporal Processing

## Table 3 of the main manuscript provides Pearson correlations between improvement outcomes for all participants (N = 64). Pearson correlations, for only participants who received AVG training (rather than all participants), are provided below.

Pearson correlational analyses indicated that flicker fusion performance at baseline (T1) significantly and negatively correlated with improvements in temporal processing following training, indicating that lower initial flicker performances were associated with greater FFT improvement following AVG training. More proficient high contrast flicker fusion scores at baseline was also positively associated with improvements in reading comprehension. Additionally, following AVG training, the amount of improvement in low contrast flicker fusion was significantly and positively correlated with reading accuracy improvements, suggesting that those who experienced the most improvement in low contrast FFT after training also experience d greater reading accuracy improvements (See Table S2).

The main manuscript also provides regressions to assess the contribution of low contrast flicker fusion performance at baseline (T1) to improvements in temporal processing following training, and to assess the contribution of post-training improvements in temporal processing to degree of improvements in reading accuracy. Similar results were found when only the AVG groups were included in these regression analyses. Lower low contrast flicker fusion scores at baseline significantly predicted greater improvement in low contrast flicker fusion performance following AVG training, explaining 55.5% of the variance in the regression model; *F* (1, 42) = 52.363, *β* = -.745, *p* < .001. Improvement in low contrast flicker fusion following AVG training was then found to be a significant predictor of improvement in reading accuracy following AVG training, explaining 11.2% of the variance in the regression model; *F* (1, 42) = 5.149, *β* = .334, *p* = .029.

| Table S2.  *Correlations between Flicker Fusion Performance and Reading Improvement Scores for AVG Participants.* | | | | | | | | |
| --- | --- | --- | --- | --- | --- | --- | --- | --- |
|  | Baseline 5% FFT (T1) | Baseline 75% FFT (T1) | Reading Accuracy Improvement | Reading  Rate Improvement | Reading Comp Improvement | Rapid Naming Improvement | 5% FFT Improvement | 75% FFT Improvement |
| Baseline 5% FFT (T1) | - | .432^**^ | -.286 | .058 | .240 | -.130 | -.745^**^ | -.018 |
| Baseline 75% FFT (T1) |  | - | -.131 | .101 | .368^*^ | -.080 | -.345^*^ | -.460^**^ |
| Reading Accuracy Improvement |  |  | - | .251 | -.145 | .024 | .334^*^ | -.018 |
| Reading Rate Improvement |  |  |  | - | .113 | -.038 | -.179 | -.122 |
| Reading Comp Improvement |  |  |  |  | - | -.219 | -.196 | -.083 |
| Rapid Naming Improvement |  |  |  |  |  | - | .099 | -.062 |
| 5% FFT Improvement |  |  |  |  |  |  | - | .240 |
| 75% FFT Improvement |  |  |  |  |  |  |  | - |
| *Note*. **p* < .05, ** *p* < .01; According to Cohen’s guidelines, *r* > 0.10, *r* > 0.30, and *r* > 0.50, represent small, medium, and large effect sizes, respectively; Improvements scores were calculated as post-training score (T2) minus baseline (T1) score; FFT = Flicker Fusion Threshold (Hz). | | | | | | | | |
